# Concerning Confidence: Serious Illness Conversations During the Medicine Acting Internship

**DOI:** 10.1089/pmr.2021.0065

**Published:** 2022-02-23

**Authors:** David Staudt, Corrie Stankiewicz, Laura E. Dingfield

**Affiliations:** ^1^ Department of Medicine, Hospital of the University of Pennsylvania, Philadelphia, Pennsylvania, USA.; ^2^ Perelman School of Medicine, University of Pennsylvania, Philadelphia, Pennsylvania, USA.; ^3^ Department of Medicine, Corporal Michael J. Crescenz VA Medical Center, Philadelphia, Pennsylvania, USA.

**Keywords:** cross-sectional survey, medical student education, serious illness conversations

## Abstract

***Background:*** Despite increased recognition that physician–patient communication represents a key competency for medical trainees, relatively little is known about student exposures to conversations about serious illness.

***Objective:*** To characterize student experiences with multiple types of serious illness conversations during their medicine acting internship (AI).

***Design:*** This is a cross-sectional survey.

***Setting/Subjects:*** Final-year medical students who had completed a medicine AI within one year at one U.S. medical school.

***Measurements:*** Exposures to and perceptions of multiple conversation domains (discussions with upset patients/families, breaking bad news, assessing code status, and conversations about limiting or withdrawing life-sustaining treatments).

***Results:*** We collected 82 survey responses (78% response rate). Students reported multiple exposures across most domains, some of which included leading conversations without supervision or formal instruction. In most domains, at least 50% of students reported confidence in their ability to lead unsupervised conversations moving forward.

***Conclusions:*** After a four-week AI, students reported multiple exposures to a variety of serious illness conversations. Some reported having these conversations without supervision. Student confidence in their ability to lead these conversations independently was higher than has been previously reported in other small studies. Further exploration is required to better understand these trends, and targeted curricular development may be indicated.

## Introduction

In the 1990s, graduating medical students reported low rates of exposure to and confidence in their ability to conduct difficult conversations across a variety of domains, particularly surrounding end-of-life care.^1^ Since then, liaison committee on medical education requirements and increasing attention to communication and palliative care topics have led to the adoption of at least some palliative care curricula across all U.S. medical schools.^2^^,^^3^ Palliative care experts have identified essential communication competencies for medical students, including giving bad news; discussing resuscitation preferences; exploring patient and family understanding of illness, concerns, goals, and values; and demonstrating a basic approach to handling emotion in patients and families facing serious illness.^4^

Furthermore, internal medicine residency program directors (PDs) have identified similar communication skills (including breaking bad news, discussing advance directives, navigating code status discussions, and communicating with difficult patients) as a high priority for incoming trainees.^5^ However, the structure and content of this curricula are variable, and current literature suggests that students continue to feel unprepared to guide code status and other conversations about serious illness as they transition to internship.^6^^,^^7^ In this context, many PDs have raised concerns about the inadequacies of the undergraduate medical education curriculum in preparing trainees for internship and residency.^5^^,^^8^

Acting internships (AIs) are increasingly standardized rotations designed to prepare students with the skills and knowledge needed to successfully transition to graduate medical education training programs. However, current curricular guidelines for medicine AIs do not address the conversations about serious illness that PDs identify as important skills for matriculating interns.^9^ Relatively little is known about student experiences with serious illness communication in the context of these rotations. Therefore, as part of a needs assessment to inform curricular development, we surveyed fourth year medical students about their experiences with a variety of serious illness conversations after completion of their medicine AI.

## Methods

This study was determined to be exempt by 45 CFR 46.104, category 2 by the University of Pennsylvania Institutional Review Board.

### Survey development

Key domains of serious illness communication important for incoming residents were drawn from consensus palliative care competencies,^4^ the internal medicine PD survey,^5^ and discussion with local communication experts. These included having conversations with upset patients and families, discussing code status, breaking bad news, and discussing limiting or withdrawing life-sustaining treatments. An electronic survey (Qualtrics, Provo, UT) was developed to evaluate student exposure to discussions in each domain. The survey included questions about the number of exposures students had to each communication domain during their AI (ranging from “0” to “4 or more”) and what roles they had in each conversation (passive observation, participation without leading, leading with supervision, and leading without supervision).

The survey included an item about whether students had received formal instruction in each domain. The survey also asked students whether they wished they had additional training before their AI for each domain, their level of confidence in each area at the end of the AI, and desired domains for additional communication training. The survey was shared with a sample of internal medicine residents to establish face and content validity. No changes were made after that review, survey structure was finalized, and the survey was distributed to participants.

### Participants and data collection

All medical students who completed a medicine AI in 2018 at the University of Pennsylvania Perelman School of Medicine (spanning three clinical sites) were surveyed (*N* = 105). Participants were contacted through e-mail with an anonymous link to the online survey form. Responses were collected over a period of eight weeks.

### Data analysis

Descriptive statistics were calculated using the total number of survey responses, including both complete and partial responses. Mean exposure estimates were calculated by assigning a value of 4 for all students who reported “4 or more” exposures to a given conversation type. Free text responses were discussed by all authors and grouped into thematic categories based on consensus decision making.

## Results

Of 105 students sent the survey, a total of 82 survey responses were submitted (77 complete; 5 partial) for a 78% response rate. Results by topic are reported hereunder, with additional detail provided in Table 1.

**Table 1.** Survey Results

	Percent exposed, % (***n***)	Mean exposure number	Reported 4+ exposures, % (***n***)	Reported formal training, % (***n***)	Passively observed, % (***n***)	Participated without leading, % (***n***)	Led with supervision, % (***n***)	Led without supervision, % (***n***)	Confident to lead independently, % (***n***)
Conversations with upset patients and families	99 (81)	3.1	48 (39)	83 (68)	41 (34)	43 (35)	56 (46)	74 (61)	74 (61)
Discussing code status	91 (75)	3.0	56 (46)	45 (37)	38 (31)	33 (27)	46 (38)	52 (43)	59 (48)
Breaking bad news	95 (78)	2.3	24 (20)	80 (66)	39 (32)	37 (30)	46 (38)	30 (25)	50 (41)
Discussing limiting/withdrawing treatments	60 (49)	1.0	6 (5)	29 (24)	37 (30)	27 (22)	17 (14)	7 (6)	16 (13)

Exposure information for serious illness conversations, including the frequency with which students encountered these conversations; what exposures they had; whether they had formal teaching around these conversations; and their confidence in their ability to have these conversations independently moving forward. Percentages are reported, with absolute number of responses included in parentheses.

### Conversations with upset patients/families

Eighty-one (81/82, 99%) students reported some exposure to conversations with upset patient/families, ranging from passive observation through unsupervised practice. Students reported a mean of 3.1 exposures to conversations with upset patients/families during their AI, with 48% (39/82) reporting “4 or more” exposures (Table 1). Seventy-four percent (61/82) of students led these conversations without supervision. At the end of their AI, 74% (61/82) of students reported confidence in their ability to have these conversations independently.

### Discussing code status

Seventy-five students (75/82, 91%) reported exposure to code status discussions. Students reported a mean of 3.0 exposures during their AI and 56% (46/82) reported “4 or more” exposures. Fifty-two percent (43/82) reported leading these conversations without supervision by the end of their AI, and 59% (48/82) were confident in their ability to discuss code status independently.

### Breaking bad news

Seventy-eight students (78/82, 95%) reported exposure to a discussion that included breaking bad news during their AI. Students reported a mean of 2.3 exposures, with 24% (20/82) reporting “4 or more exposures” to these conversations. Thirty percent (25/82) led conversations that included breaking bad news without supervision and 50% (41/82) were confident in their ability to break bad news independently after their AI.

### Discussing limiting or withdrawing potentially life-sustaining treatments

Forty-nine students (49/82, 60%) were exposed to conversations that included discussion of limiting or withdrawing potentially life-sustaining treatments. Students reported a mean of 1 exposure, whereas 6% (5/82) had “4 or more” exposures during their four-week AI. Seven percent (6/82) reported leading these conversations independently. At the end of the AI, 16% (13/82) of students were confident in their ability to independently discuss limiting or withdrawing potentially life-sustaining treatments with patients.

### Open-ended responses

Students identified multiple other challenging communication domains they encountered during the AI (Table 2). A total of 11 students (13%) submitted free text responses. Responses were grouped into four categories based on content: conversations around prognosis and goals of care (five comments), managing team dynamics (two comments), discussing medical errors (two comments), and miscellaneous (two comments).

**Table 2.** Free-Text Responses to Open-Ended Questioning About Whether There Were Additional Areas Students Wished They Had Communication Training in Before Their Acting Internship

Theme	Comments
Conversations around prognosis and goals of care	“Goals of care conversations for patients with [end-stage renal disease]”
	“Patients who are indecisive about treatment…whose actions are in conflict with their stated goals…”
	“Speaking and conveying diagnosis and prognosis to non-English speaking patients”
	“[Exposure to difficult conversations] really depends on [our own] training. In the past, I felt like doctoring tried to have [these conversations] but they weren't effective and felt preachy”
	“Discussing situations in which there are no opportunities to intervene in the course of a chronic disease”
Managing team dynamics	“How to decide how much of the/your plan to share with the patient, given that you are their primary source of information and they want to know what's going to happen but also that the rest of the team may change your plan significantly”
	“Dealing with defensive supervisors. Patient communication was often handled extremely poorly and patients were left feeling lied to and mistreated, but any suggestion that we could be doing better was taken as a personal offense and duly punished”
Discussing medical errors	“Anything that might have legal implications…”
	“How to handle a patient being upset about a mistake that you or another provider made”
Miscellaneous	“How to handle personal verbal attacks”
	“Conversations about substance use/misuse”

## Discussion

In this single-center study, students described their experiences with serious illness conversations during a medicine AI. Some students reported formal training in each serious illness conversation domain. Reports of formal training were lowest for code status discussions and conversations about limiting/withdrawing life-sustaining treatments. Students reported clinical exposures in all conversation domains. Despite low mean number of exposures, a majority of students led conversations without supervision and were confident in their abilities to have conversations with upset patients/families, about code status, and breaking bad news independently after their AI.

Our results have several limitations. First, the single-center nature may limit generalization—that said, literature has noted potential inadequacies in this area across multiple institutions.^3^^,^^6^ Second, survey responses are susceptible to recall bias, and many students completed their AI months before survey completion that may exaggerate this phenomenon. Although we defined formal training as “lectures, role-play, or standardized patient encounters,” the variability of student responses at a single institution suggests they may have differing perceptions of what constitutes formal training.

We do not have data about confidence levels before the medicine AI, and although confidence seems to be highest in the most frequently encountered domains, an understanding of pre-existing confidence levels would help better characterize these trends. Lastly, we assigned a numerical value of “4” for all answers of “4 or more” for the purposes of calculating mean exposures, which may under-represent the frequency at which students encountered these conversations.

Despite these limitations, our study raises several interesting points for discussion. Prior studies suggested that significant minorities (∼40%) of students go through major portions of medical training with limited exposure to conversations about prognosis, breaking bad news, code status, and end-of-life care.^1^^,^^10^ We found that over the course of the medicine AI, most students were exposed to each of these conversations and many reported multiple exposures.

In contrast with recent literature,^6^ students in our survey endorsed relatively high degrees of confidence in their abilities to have independent discussions across the spectrum of the communication domains. Students were least confident for domains in which they lacked formal training and had little exposure (discussions of limiting/withdrawing life-sustaining treatments) and most confident for domains in which they reported both training and frequent exposure (talking with upset patients/families). Although only a minority of students reported formal training in code status discussions, these were relatively frequently encountered in the clinical learning environment.

The majority of students reported that they led code status conversations without direct supervision despite lacking formal training, and most students were confident in their ability to independently lead these conversations at the conclusion of the AI. This gap between training experience and confidence may be consistent with literature that suggests that among residents, confidence surrounding serious illness communication increases over the course of multiple exposures but does not correlate with competency.^11^^,^^12^ Our data suggest that confidence in serious illness communication may emerge earlier in training than previously recognized, particularly in the domain of code status conversations, and could represent emergence of a confidence-skill gap during the AI, or earlier.

In summary, students were exposed to serious illness conversations over the course of the AI and report conducting these conversations without supervision. Confidence in their ability to independently lead these conversations was highest for those conversations that were most frequently encountered and for which they had received some training. Future study is needed to better understand patterns across institutions, to explore the possibility of a confidence-skill gap emerging among students in some communication domains, and to determine whether closer supervision or targeted curriculum development is needed to increase student competency in these areas to better prepare them for transition to residency.

## Abbreviations Used

AI: acting internship
PDs: program directors
